# The clinical meaning of external cervical resorption in maxillary canine: transoperative dental trauma

**DOI:** 10.1590/2176-9451.19.6.019-025.oin

**Published:** 2014

**Authors:** Alberto Consolaro, Mauricio de Almeida Cardoso, Carolina Dornelas C. M. de Almeida, Ingrid Araújo Oliveira Souza, Leopoldino Capelloza

**Affiliations:** 1 University of São Paulo, School of Dentistry, Postgraduate Program, Ribeirão Preto, Full professor, School of Dentistry - University of São Paulo/Bauru. Professor, Postgraduate Program, School of Dentistry -University of São Paulo/ Ribeirão Preto; 2 Sacred Heart University, Professor, Undergraduate and Postgraduate (Master's degree in Orthodontics) Programs, Sacred Heart University (USC); 3 Ingá College, Postgraduate student in Orthodontics, Ingá College (UNINGÁ); 4 College of Dentistry, Bauru, PhD resident in Stomatology, College of Dentistry - Bauru/USP; 5 Sacred Heart University, Professor, Undergraduate and Postgraduate (Master's degree in Orthodontics) Programs, Sacred Heart University (USC)

**Keywords:** External Cervical Resorption, Orthodontic traction, Alveolodental luxation, Dental trauma

## Abstract

External Cervical Resorption in maxillary canines with pulp vitality is frequently associated with dental trauma resulting from surgical procedures carried out to prepare the teeth for further orthodontic traction. Preparation procedures might surgically manipulate the cementoenamel junction or cause luxation of teeth due to applying excessive force or movement tests beyond the tolerance limits of periodontal ligament and cervical tissue structures. Dentin exposure at the cementoenamel junction triggers External Cervical Resorption as a result of inflammation followed by antigen recognition of dentin proteins. External Cervical Resorption is painless, does not induce pulpitis and develops slowly. The lesion is generally associated with and covered by gingival soft tissues which disguise normal clinical aspects, thereby leading to late diagnosis when the process is near pulp threshold. Endodontic treatment is recommended only if surgical procedures are rendered necessary in the pulp space; otherwise, External Cervical Resorption should be treated by conservative means: protecting the dental pulp and restoring function and esthetics of teeth whose pulp will remain in normal conditions. Unfortunately, there is a lack of well-grounded research evincing how often External Cervical Resorption associated with canines subjected to orthodontic traction occurs.

## Abstract (pt)

A reabsorção cervical externa em caninos superiores com vitalidade pulpar em sua quase totalidade está associada a traumatismo dentário decorrente de procedimentos cirúrgicos associado à preparação desse dente para ser tracionado ortodonticamente. Nessa preparação pode se manipular cirurgicamente a junção amelocementária ou luxar o dente com forças excessivas ou com testes de movimentação além dos limites de tolerância estrutural do ligamento periodontal e tecidos cervicais. A exposição dentinária na junção amelocementária é o estopim para se iniciar uma reabsorção cervical externa a partir de uma inflamação induzida na região seguida de reconhecimento antigênico das proteínas dentinárias. A reabsorção cervical externa é indolor, não induz pulpites e tem uma evolução lenta. Em geral, a lesão está associada e recoberta por tecidos moles gengivais que mantêm, por longos períodos, os aspectos clínicos normais, induzindo diagnósticos tardios, quando o processo se aproxima dos limites pulpares. O tratamento endodôntico está indicado apenas em função de procedimentos operatórios que se fazem necessários no espaço pulpar; caso contrário, a reabsorção cervical externa deve ser tratada de forma conservadora, protegendo a polpa dentária e restaurando a função e estética do dente que permanecerá com sua polpa normal. Infelizmente, não sabemos, com base em pesquisas de casuísticas bem estabelecidas, qual é a frequência da reabsorção cervical externa associada a caninos ortodonticamente tracionados.

A simple case may provide the orthodontic science with fruitful ideas. Insights are recurrent as the reality is dynamic: the needs of the past are no longer the needs of the present. In the health sciences, scientific information acquires historical value within five years, as they are owned by public domain and replaced by new ones. In some fields of study, such as chemistry, physics and information science; knowledge and reality are updated so fast that paper journals cannot follow their pace: Within six years, up-to-date knowledge completely changes these fields of study. Likewise, social changes are so increasingly fast that the current orthodontic reality is no longer comparable to that of five years ago. 

In the present study we report a case that serves as an example not only to highlight the clinical meaning of External Cervical Resorption, but also to discuss the parameters established for differential diagnosis and some preventative measures that should be taken to prevent the condition from happening in surgical practice!

## A case report: An insight

**Figure 1.**
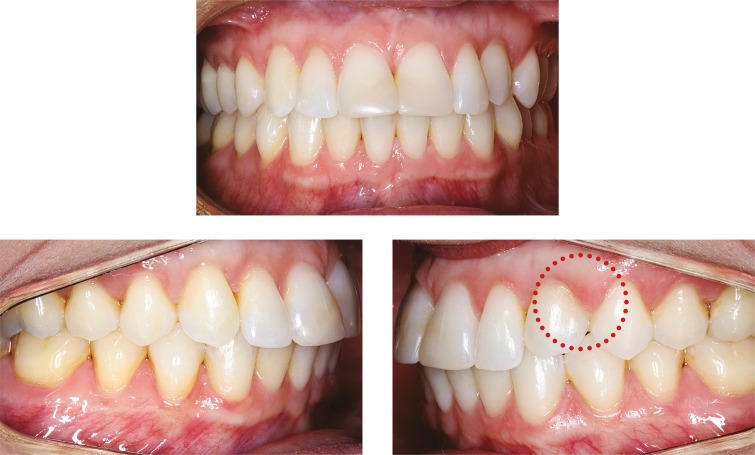
Dental and gingival condition of patient with External Cervical Resorption in left maxillary canine (circle). Note a discrete red area in the gingival papilla overlaying the region of External Cervical Resorption.

After seven years of orthodontic treatment, a 27-year-old patient (Fig 1) presented with chief complaint of slight gingival alteration and unpleasant taste in the cervical region of painless left maxillary canine.

**Figure 2.**
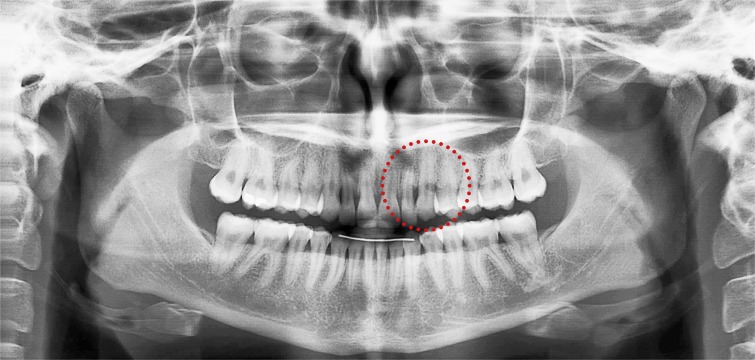
External Cervical Resorption in left maxillary canine (circle) revealed by panoramic radiograph. The examination suggests advanced-stage lesion based on the dimensions required by this type of exam to identify a case of tooth resorption.

**Figure 3.**
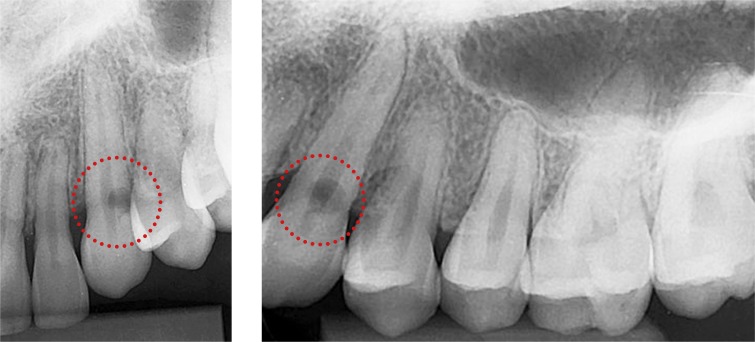
External Cervical Resorption in left maxillary canine (circle) revealed by periapical radiograph. Note preserved pulp threshold despite large dimensions.

**Figure 4.**
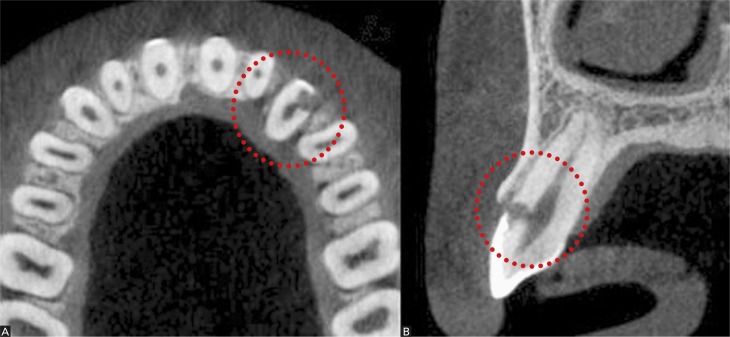
External Cervical Resorption in left maxillary canine (circle) revealed by two major tomographic slices: Axial (A) and sagittal (B). Note that CT scans do not reveal preserved pulp threshold due to the extremely delicate dentin wall remaining around the dental pulp at the site of External Cervical Resorption.

**Figure 5.**
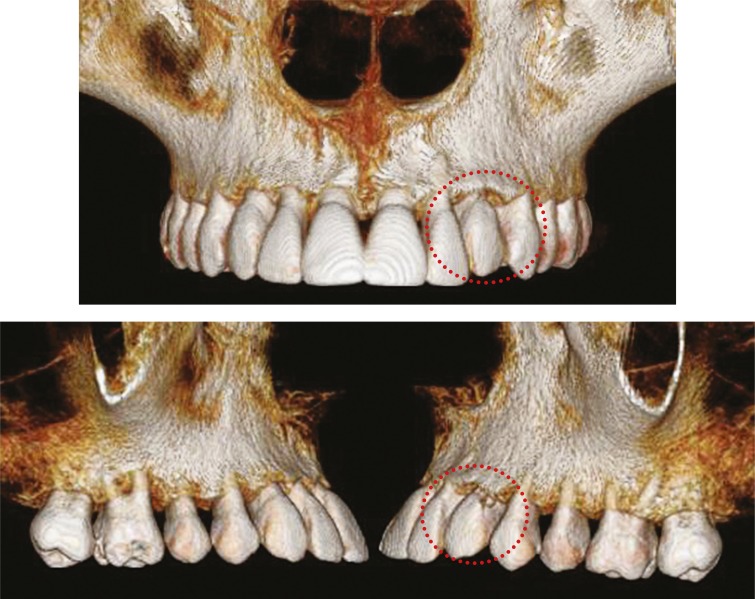
External Cervical Resorption in left maxillary canine (circle) revealed by two major tomographic slices: Axial (A) and sagittal (B). Note that CT scans do not reveal preserved pulp threshold due to the extremely delicate dentin wall remaining around the dental pulp at the site of External Cervical Resorption.

Panoramic (Fig 2) and periapical (Fig 3) radiographs revealed radiolucent lesion in the cervical region of the buccodistal surface of the canine. The condition was confirmed by tomographic slices (Fig 4) and 3D images (Fig 5). The lesion was deep and located near pulp threshold. Nevertheless, gingival tissues were close to normality in spite of discreet red macule in the papilla overlaying the resorptive lesion (Fig 1).

## Identifying the cause

The patient reported having undergone orthodontic traction seven years before without surgical luxation. The patient also stated not remembering any episode of dental trauma in the affected tooth. 

## Diagnostic discussion

In the vast majority of cases, even those requiring CT scans, the pulp threshold remains preserved in the areas corresponding to External Cervical Resorption lesion. The clasts absorbing hard tissues do not attach to soft tissues, as it is the case of predentin. Whenever they meet it, they move laterally and subsequently mineralize, thereby preserving the integrity of pulp space and pulp for long periods of time.

In many cases, however, pulp threshold is extremely thin and little mineralized, which results in lack of imaginologic signs as in the case presented herein. Considering that the tooth was painless and responded positive to sensitivity testing, it is reasonable to assume the existence of a preserved pulp threshold even if by a thin dentin or predentin layer. Even the most intense and deep resorptive processes do not lead to pulpitis or inflammation of dental pulp. As a result, they do not evince any painful symptoms.

## Adopted protocol: Conservative treatment with pulp protection

**Figure 6.**
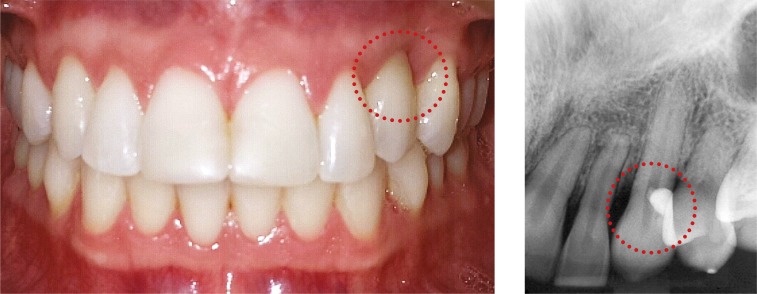
Dental and gingival condition of patient with External Cervical Resorption in left maxillary canine (circle) three months after restorative treatment onset.

**Figure 7.**
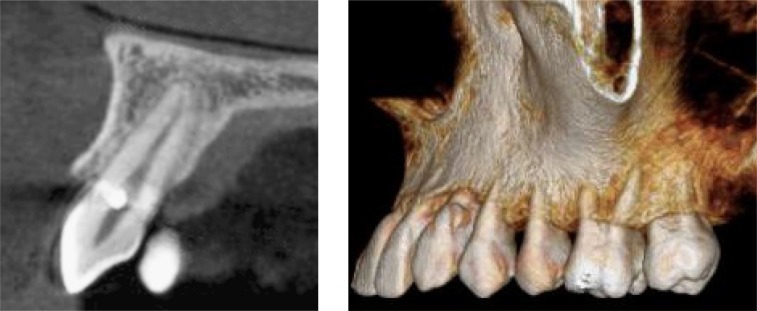
Sagittal tomographic slice and 3D CT scan of External Cervical Resorption in left maxillary canine (circle) three months after restorative treatment onset.

In view of normal gingival conditions and proved pulp vitality, and after identifying the cause that no longer acted, but nurtured a process that needed to be interrupted, the protocol of choice was functional and esthetic restoration aimed at preserving the pulp and covering it with calcium hydroxide-based material. Three months have passed after the procedure was carried out. Patient's tooth presents in normal conditions in terms of esthetics and function without symptoms or color alterations (Figs 6 and 7).

## Differential diagnosis

*Cervical caries:* It usually causes exposure of the root surface as a result of gingival recession, with dark and irregular decay advancing towards the tooth crown. In cases of External Cervical Resorption, decay is not dark and is usually covered or hidden by gingival tissues which overlays surface mineralized structures.

*Abrasion: *A V-shaped lesion with regular surface and ridge exposed to the oral cavity. When in the cervical region, it is generally associated with improper tooth brushing. It is hardly ever found in one tooth only.

*Abfraction:* Cervical enamel microfractures with small, hard-wall lesions without dark pigmentation. Lesions are exposed to the oral cavity as a result of gingival recession.

*Erosion: *Lesions exposed to the oral cavity caused by chemical dissolution not of bacterial origin. Lesions are normally saucer-shaped and smooth, present a direct cause-and-effect relationship and affect several teeth instead of just one. 

## What is the clinical meaning of External Cervical Resorption?

All cases of External Cervical Resorption in teeth with pulp vitality are caused by dental trauma, whether accidental or surgically induced.

Dental trauma, particularly mild ones, might cause focal lesions to connective tissue related to the cervical region of the root and its gingival connective attachment. That is where the cementoenamel junction is. In this region, along the tooth border, the cementum might overlap the enamel, cementum and enamel might abut each other or there might be a space or a gap between the enamel and the cementum where the dentin is exposed to the gingival connective tissue. These three variations in the relationship among enamel, cementum and dentin exist in all permanent and deciduous human teeth, and vary according to the site of the tooth border examined.

Provided that it occurs without migration of junctional epithelium, gingival tissue inflammation ends up exposing the dentin in the gaps to antigen recognition/immunologic cells which recognize kidnapped or hidden dentin proteins.

With an exudate rich in enzymes, gingival inflammation dissolves the gelled extracellular matrix of the connective tissue which protects or tenuously covers the dentin exposed in the gaps, thereby preventing contact between the dentin and protein recognition macrophages. In the event of antigen recognition of dentin proteins, immunologic response promotes dentin removal, thereby leading to Enamel Cervical Resorption.

Cases in which the patient does not recall dental trauma are usually cases of dental concussion characterized by not promoting increase of tooth mobility. In these cases, should there be any symptoms, they are mild and only last for a few hours. This type of mild dental trauma might be caused as a result of bumping against toys, punch, body contact during sports practice, falls and other daily activities. After a few hours, people rarely remember about the concussion.

As for erupted maxillary canines, the consequences of concussion might not be as serious due to teeth extensive root base at periodontal attachment. Concussion alterations only occur in maxillary canine when they are highly severe. Nevertheless, they are more common in central and maxillary lateral incisors.

Cases of External Cervical Resorption in maxillary canines should be immediately related to transoperative dental trauma instead of accidental trauma:

**1. During bracket/button bonding procedures or enamel perforation.** During bracket/button bonding procedures or enamel perforation, surgeons usually remove the pericoronal follicle up to its cervical attachment. As a result, they inevitably end up manipulating the cementoenamel junction in an improper way; thereby directly exposing the dentin to macrophages in the cervical connective tissue. The process of External Cervical Resorption starts slowly and might only be revealed by imaging or clinical examinations when the tooth is already present in the dental arch. 

**2. Alveolodental luxation surgically assisted or not by the orthodontist.** During procedures of surgically-assisted luxation or manual/instrument movement tests, the tooth undergoes **significant** movement/displacement. For 0.25-mm thick periodontal tissue, these movements represent a true transoperative dental trauma and lead to similar consequences as those caused by accidental trauma. The process of External Cervical Resorption starts slowly and might only be revealed by imaging or clinical examinations when the tooth is already present in the dental arch. 

## How can we prevent External Cervical Resorption in maxillary canines potentially subjected to orthodontic traction?

In general terms, External Cervical Resorption is prevented by avoiding accidental dental trauma. Although the use of a mouthpiece is not widely spread in Dentistry and within the overall population, it should be mandatory during sports practice and leisure activities. Most dental professionals are unaware of mouthpieces and the benefits provided by their routine use in certain situations.

In the event of External Cervical Resorption induced by transoperative dental trauma, some important measures must be taken: 

## **1.** **During bracket/button bonding procedures or enamel perforation **carried out for orthodontic traction purposes, the orthodontist must interact with the surgeon so as to:

**a) While opening up a space or "gap" **in the pericoronal follicle so as to have access to the enamel, do not remove soft tissues up to the cervical region where they are attached. Leave a 1 to 2-mm strip of soft tissue in order to avoid unwanted manipulation of the cementoenamel junction.

**b) During enamel acid etching**: should it be the technique of choice to prepare the surface receiving resin and bracket/button, avoid material overflow in the cervical region as it might act on the cementoenamel junction and, as a result, expose it and increase the size of dentin gaps. 

2. In the event of surgically-assisted luxation for orthodontic traction, some important measures must be taken together with the surgeon:

a) Surgically-assisted luxation should not be carried out **unless it is requested by the orthodontist.** It should be evinced that orthodontic traction of unerupted teeth without alveolodental ankylosis does not require previous luxation. **Emphasize:** Surgically preparing a tooth for orthodontic traction is one procedure, surgically-assisted alveolodental luxation is something completely different, carried out in a distinct moment.

b) Whenever surgically-assisted luxation is **recommended by the orthodontist**, the surgical procedures carried out for alveolodental luxation should be as delicate as possible particularly because the periodontal ligament is on average 0.25-mm thick, as thin as a paper sheet.

## c)** Movement/displacement testing **during alveolodental luxation surgery should be extremely delicate so as to avoid dental trauma of which consequences include External Cervical Resorption.

## Final considerations

This case report motivated us to question the following in epidemiological terms:

" How many cases (%) of teeth subjected to orthodontic traction present External Cervical Resorption as a consequence of the therapy of choice?
" How many of these cases (%) are associated with surgical procedures of bracket/button placement or enamel perforation for orthodontic wire installation, only?
" How many of these cases (%) are associated with procedures of surgically-assisted alveolodental luxation, only?

There are significant case reports allowing these data to be analyzed so as to enlighten the orthodontic practice! May these insights disturb all and let research begin.
